# Machine Learning Inversion of Layer-Wise Plasticity and Interfacial Cohesive Parameters in Multilayer Thin Films

**DOI:** 10.3390/ma18214976

**Published:** 2025-10-31

**Authors:** Baorui Liu, Shuyue Liu, Kaiwei Xing, Zhifei Tan, Jianru Wang, Peng Cao

**Affiliations:** 1 College of Architecture and Civil Engineering, Beijing University of Technology, Beijing 100124, China; 2 Science and Technology on Reliability and Environment Engineering Laboratory, Beijing Institute of Structure and Environment Engineering, Beijing 100076, China; 3 Department of Civil and Environmental Engineering, The Hong Kong Polytechnic University, Kowloon, Hong Kong; 4 Academy of Aerospace Solid Propulsion Technology, Xi’an 710025, China

**Keywords:** machine learning, nanoindentation, cohesion parameter, parameter back analysis

## Abstract

This study proposes a fast material parameter evaluation method for multilayer thin-film structures based on machine learning technology to solve the problems of long time and low efficiency in the traditional material parameter inversion process. Nanoindentation experiments are first conducted to establish an experimental basis across film stacks. A two-dimensional elasto-plastic model of the indentation process is then built to generate a large set of load–depth curves, which serve as training data for a machine learning model. Trained on simulated curves and validated against measurements, the model enables fast inverse identification of layer-wise plastic parameters and interfacial cohesive properties. The experimental results show that the method has high accuracy and efficiency in the inversion of interlayer cohesion parameters, and the correlation coefficient R^2^ is 0.99 or more. Compared with traditional methods, the pipeline supports batch analysis of multiple datasets and delivers parameter estimates within 1 h, substantially shortening turnaround time while improving result reliability. This method can not only effectively solve the challenges faced by traditional material evaluation, but also provide a new and effective tool for the performance evaluation and optimization design of multilayer thin-film materials. It has broad application prospects and potential value.

## 1. Introduction

Multilayer thin-film structures have received extensive attention in microelectronics, optics, and thin-film coatings because of their excellent physical properties and broad applicability in modern electronic devices and thin-film materials [1,2,3]. In multilayer film structures, the mechanical properties and interfacial adhesion properties of multilayer film structures directly determine their reliability and durability in various applications [4,5]. Therefore, an in-depth study of film material parameters and interfacial adhesion properties is of great significance for optimizing the design of multilayer films and improving their application properties.

Due to the complex interlaminar interfaces of multilayer films and the heterogeneity of each layer of materials, it is necessary to study the interface adhesion properties of multilayer films, making it difficult to accurately assess their overall mechanical properties by conventional mechanical testing methods such as tensile, compression, bending, shear, and fatigue tests [6]. In addition, traditional mechanical testing methods are often destructive and require the Preparation of specimens of standard sizes and shapes. At the same time, multilayer thin-film structures tend to be in the range of nano-to-micron-scale thickness. The thickness of multilayer thin-film structures is often in the range of nano-to-micron-scale thickness, making it challenging to meet the sample preparation requirements for these tests [7]. In recent years, the nanoindentation test, as a high-precision characterization technique of the mechanical properties of materials, has gradually become an important tool for studying the mechanical properties of multilayer film structures [8,9,10]. Especially in the study of multilayer thin-film structures, nanoindentation tests can effectively measure the properties of materials at different levels, and provide important experimental data for further understanding the interface effect and interlaminar mechanical behavior of thin films [11,12,13]. Therefore, in this paper, nanoindentation technology will be used to obtain material parameters to evaluate the mechanical properties of multilayer films more accurately.

However, the data obtained by nanoindentation tests often contain a large amount of nonlinear information, which makes traditional evaluation methods face significant challenges in extracting material parameters [14,15]. During indentation testing, conventional nanoindentation typically provides only the overall elastic modulus, making it difficult to directly resolve the plastic yield stress of individual layers and the interlayer bonding parameters [16,17,18]. Therefore, researchers often use some auxiliary tools to analyze the mechanical response of nanoindentation experiments, in which finite element (FE) simulation is widely used [15]. Finite element simulation can simulate the indentation process at the macro and micro levels, thereby extracting the mechanical properties of the material [19]. However, finite element simulation relies on a large amount of experimental data and consumes a large amount of computational resources when performing material parameter analysis [20,21,22].

In recent years, with the rapid development of machine learning technology, its application potential in materials science has gradually received extensive attention [23,24,25]. By learning and training on a large amount of data, machine learning can automatically identify and extract the complex nonlinear relationship between the microstructure and mechanical properties of materials to achieve efficient prediction. Yang et al. [26] employed a back-propagation (BP) neural network to identify the elastic–plastic parameters of the ceramic matrix composite. The results show that the error value of the neural network is controlled within 5%. Han et al. [27] used an artificial neural network to evaluate the thin-film metallic glass, and the experimental results show that the error value of the neural network is less than 3%. In addition, machine learning has been widely used in many fields such as alloys [28], nanomaterials [29], and polymers [7]. However, the application of nanoindentation data analysis in multilayer films has not been reported.

Based on the above background, this study aims to explore the nanoindentation test of multilayer thin-film structure and its material parameter evaluation method, focusing on applying machine learning technology to realize the rapid reverse analysis of material parameters. Specifically, a two-dimensional multilayer film model was first constructed in Marc Software (version 2024.2) [30], and the specimens’ material parameters and interfacial bonding properties were characterized by the elastic–plastic model and the cohesive zone model to generate the required dataset. Subsequently, a machine learning model suitable for multilayer thin-film nanoindentation data was developed on the ODYSSEE platform (version 2024.2), and the model was trained and optimized in combination with experimental data. Finally, by comparing the model prediction results with the experimental curves, the multilayer film’s elastic-plastic parameters and cohesive parameters are quickly inverted. Compared with the traditional inverse analysis method, the proposed method can obtain the material parameters more quickly and accurately, significantly improving the evaluation efficiency and reliability of the results. Additionally, it overcomes the longstanding limitation of conventional nanoindentation in directly resolving plastic parameters and interfacial properties. In addition, this method has a high degree of compatibility and can meet the development needs of feature analysis based on big data in the future.

## 2. Experimental Methods

### 2.1. Sample Preparation

**Figure 1:**
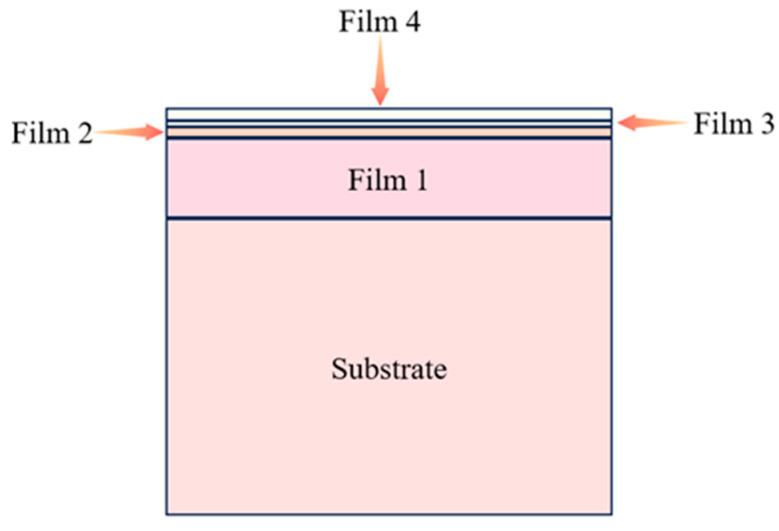
Schematic diagram of the thin-film structure.

In this experiment, four-layer thin-film samples, sourced from Beijing, China, were selected and subjected to high-temperature nanoindentation tests. The properties of the samples were tested at four temperatures: 25 °C, 200 °C, 400 °C, and 600 °C. The composition of each layer of each sample is detailed in Table 1, and the thin-film structure is shown in Figure 1. All samples were prepared by magnetron sputtering with a standard deposition process to ensure good adhesion and consistent physical properties between the layers.

### 2.2. Nanoindentation Test

#### 2.2.1. Experimental Testing Process

All specimens were tested using a high-temperature nanoindenter [31,32], which integrates precision drive, detection, position switching, temperature control, and signal processing technologies. The instrument consists of a rigid vibration isolation unit, alignment and positioning unit, precision loading unit, eccentric rotation and point-switching unit, precision sensing unit, signal processing unit, high-temperature control module, and water-cooling circulation system. It has a maximum heating temperature of 1000 °C, a heating rate range of 0.5 °C/min to 30 °C/min, and a temperature control accuracy of ±2 °C, ensuring stable and accurate testing under elevated temperatures. Before each test, the indenter tip was kept in the temperature-controlled furnace chamber for sufficient thermal equilibration to ensure uniform heating and minimize thermal expansion mismatch between the tip and the specimen. In addition, the instrument was professionally recalibrated by certified technicians prior to testing to ensure the accuracy of the tip area function and eliminate errors due to thermal drift or mechanical misalignment.

**Figure 2:**
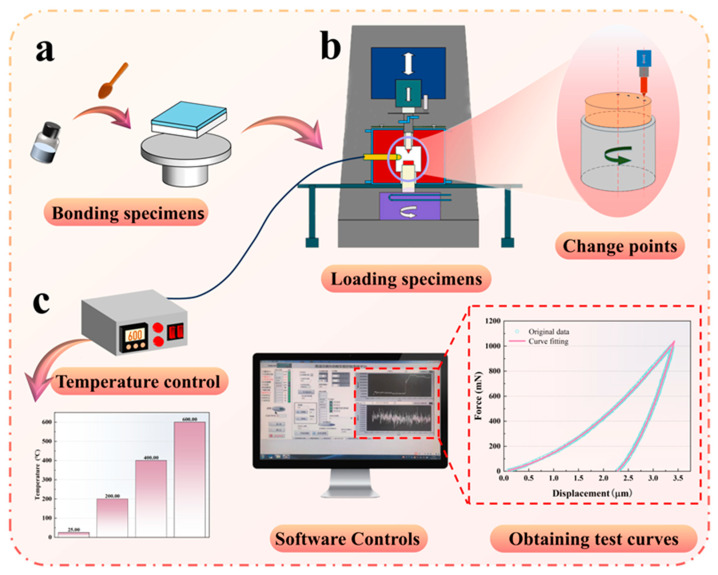
Nanoindentation test process: (**a**) bonding specimens, (**b**) testing process, (**c**) test control and curve acquisition.

The detailed test procedure is shown in Figure 2. First, each specimen was fixed onto a ceramic base using a high-temperature adhesive to prevent uneven contact or slippage between the specimen and the base during loading, thereby ensuring the accuracy of the test results. After fixation, the specimen was left to stand for 4 h until the adhesive was fully cured. Subsequently, the specimen was mounted into the nanoindenter, and its rotation was adjusted to allow indentation testing at as many locations as possible. Prior to each indentation, a thermal equilibration period of 1 h was applied to ensure temperature stability of both the specimen and indenter tip.

During indentation, the load was applied in two stages: a 20 s loading segment followed by a 20 s unloading segment. The maximum indentation depth was limited to 0.004 mm (4 µm). In order to improve the reliability and accuracy of the data, three groups of data acquisition were repeated at each test point. During the test, the indentation position was changed by rotating the specimen to ensure the data coverage of different regions and reduce the influence of local stress concentration on the results.

#### 2.2.2. Nanoindentation Analysis Method

Instrumented nanoindentation continuously records the load–penetration depth (P–h) curve during a single loading–unloading cycle. Mechanical parameters such as hardness and the (reduced) elastic modulus can be obtained from this curve. The Oliver–Pharr method is the most widely used analysis framework, and the governing equations are given below.

(1) $$H=\frac{P_{max}}{A}$$

(2) $$\frac{1}{E_{r}}=\frac{1-\nu^{2}}{E}+\frac{1-\nu_{i}^{2}}{E_{i}}$$

where *H* is the indentation hardness, *A* is the contact area, and *P_max_* is the maximum load. *E_i_* and $\nu_{i}$ denote the Young’s modulus and Poisson’s ratio of the specimen, respectively, while *E_i_* and $\nu_{i}$ are those of the indenter. In this study, a diamond indenter was used with *E_i_* = 1141 GPa and $\nu_{i}$ = 0.07.

## 3. Parameter Back Analysis Method

**Figure 3:**
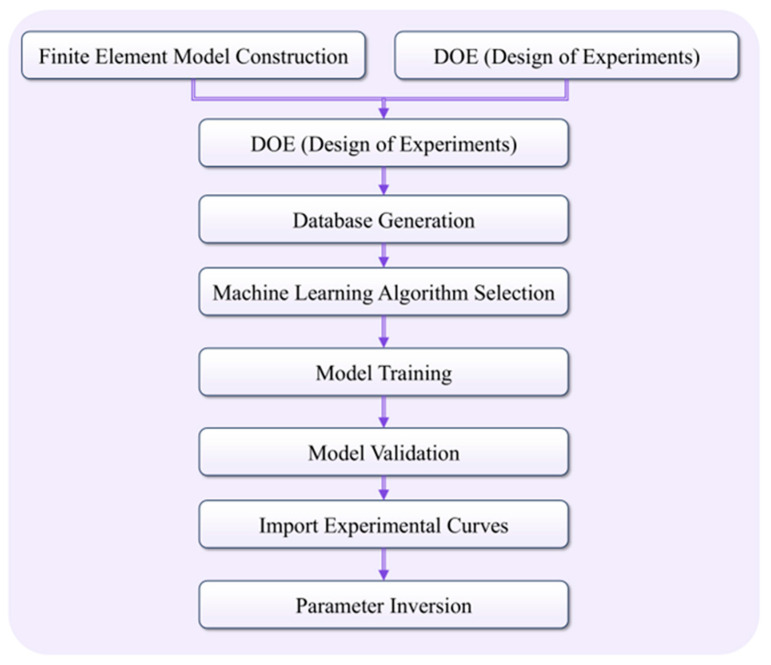
Workflow for inverse parameter identification.

This section outlines the workflow for inverse identification of interfacial cohesive parameters and layer-wise plastic properties in multilayer thin films. We first establish a finite-element model, followed by a machine learning stage. Using Design of Experiments (DOE), a dataset of nanoindentation load–depth curves is generated; the curves are then compressed via reduced-order modeling with time- and frequency-domain dimensionality reduction to capture the complex mapping between indentation responses and material parameters [33], as illustrated in Figure 3. The following subsections detail the construction of the FE model and the training procedure of the machine learning model.

### 3.1. Finite Element Modeling

#### 3.1.1. Geometric Modeling and Meshing

Because of the high computational complexity and massive resource consumption of the three-dimensional model, this study refers to the method of Zhou to improve the computational efficiency and ensure the operability of the model [30]. It analyzes the characteristics of the three-dimensional model, which is simplified into a two-dimensional axisymmetric model in Marc Software (version 2024.2). This simplification not only significantly reduces the computational time and resource consumption but also can still meet the research needs in preserving key physical features and maintaining simulation accuracy, providing a reliable basis for subsequent analysis [34,35].

In this calculation, the nanoindentation experiment is regarded as a hardness test with known loading force and displacement, and the microprobe is used to indent the sample’s surface. By making the projected area of the indenter on the specimen equal, the equivalent half-cone angle α = 70.3° [36] of the indenter (a = 24.56 h^2^) is obtained, which is equivalent to the conical indenter (A = πh^2^tan2θ); thus, the problem is simplified to a spatial axisymmetric problem. In addition, because the INDENTER used in this experiment is a diamond indenter, its modulus is much higher than that of the test material, and the final post-processing analysis only needs to extract the load and displacement data on the indenter; therefore, assuming that the indenter does not deform, it is set as a rigid body [37]. The model’s overall size is set to 0.2 mm × 0.2 mm, and the indenter size is set to 0.06 mm. The specific size of each layer model is shown in Table 2, and the simplified model is shown in Figure 2.

**Figure 4:**
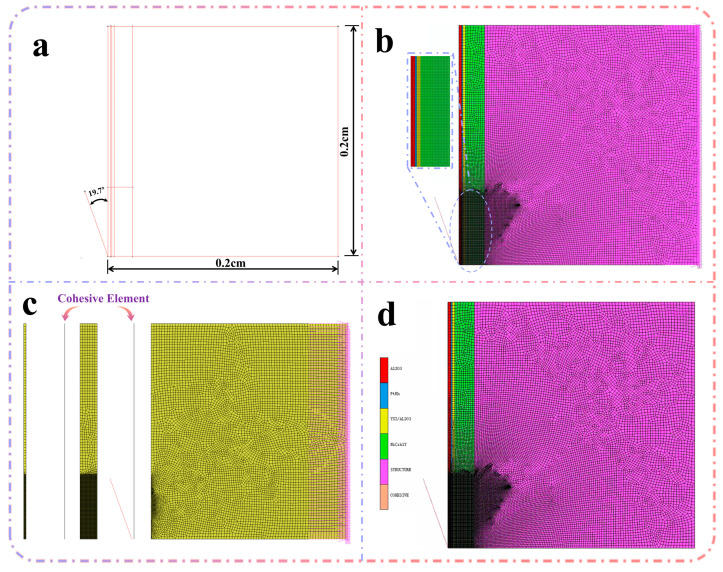
Modeling process: (**a**) geometric modeling, (**b**) grid division, (**c**) insertion of cohesion modules (the grid color is the original grid color), (**d**) giving material properties (the grid color corresponds to the color of the respective material).

After the model was established, the indenter speed was set to 0.004 mm/s, and then the indenter retreated to the starting position. In this calculation, the indenter is locally refined, the minimum mesh size is 0.0004 mm, the maximum mesh size is 0.0025 mm, the mesh type is quadrilateral unit, the total number of mesh units is 25180, and the mesh model is shown in Figure 4.

The input material parameters used in this model are listed in Table 3. However, the layer-wise yield stresses and interfacial cohesive parameters still require identification; the corresponding models are presented below.

When the material undergoes plastic deformation, large local strains may occur, and using only the initially generated mesh for the entire analysis would lead to significant computational errors. Therefore, in this study, the adaptive remeshing function in Marc was employed to automatically regenerate the mesh according to the updated geometry during the simulation process. The front-quadrilateral remeshing method was adopted to ensure element quality and numerical stability.

The interfaces between the film layers were defined as bonded contacts, while the interface between the film and the indenter was modeled as a frictional contact with a friction coefficient of 0. Additionally, all nodes on the right boundary were assigned fixed constraints to prevent rigid body motion and ensure boundary stability.

#### 3.1.2. Cohesion Model

**Figure 5:**
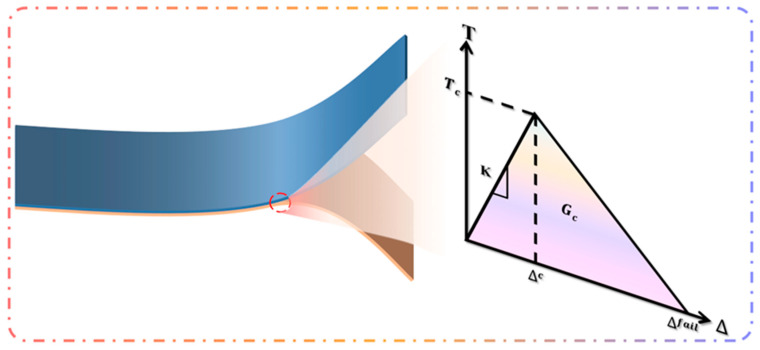
Stress principal relationship diagram for cohesive units.

Adding cohesive elements between layers is the most commonly used method in characterizing interfacial adhesion properties of thin-film structures. A cohesive element, namely a cohesive element, simulates a two-dimensional crack by presetting the crack edge and adding a layer of cohesive element with zero thickness to the predicted crack area. In MARC, the bilinear cohesive model is mainly used to simulate the cracking behavior [38]. The most important parameters in the model are the Cohesive Energy (*G_C_*), the Critical Opening Displacement (*∆_C_*), and the Maximum Opening Displacement (*∆_fail_*). The constitutive relations for the stress units are shown in Figure 5, where T_C_ is the normal phase traction force corresponding to *Δ_C_*.

(3) $$\Delta^{\mathrm{fail}}=\frac{2G_{C}}{T_{c}}$$

To accurately capture the interfacial debonding behavior between adjacent film layers, zero-thickness cohesive elements were inserted at each film interface. The element type 190 was employed to simulate the cohesive zone, enabling the model to represent progressive interfacial damage and separation under loading.

#### 3.1.3. Von-Mises Yield Criterion

In this finite element model, it is necessary to select the appropriate material model for the material, and the material model selected is the elastic–plastic model. The elastic–plastic model can more realistically simulate the behavior of thin films under large deformation and complex stress states; capture the yield, hardening, and plastic flow characteristics of materials; and thus improve the simulation accuracy, resulting in a more accurate prediction of the performance of multilayer thin-film structures, thus providing more reliable data support for analysis and optimization [39,40,41].

In order to simulate the plastic behavior of materials, the Von Mises yield criterion is used as the plastic model. The Von Mises yield criterion is a widely used yield criterion in material science and engineering, which predicts the yield behavior of materials under complex stress states by calculating Von Mises stresses. This criterion is especially suitable for isotropic materials and can accurately reflect the yield characteristics of metals and other materials [42,43]. The Von Mises yield criterion is advantageous in analyzing the nanoindentation model for multilayered thin-film structures [44]. The experimental data show that the Von Mises criterion agrees with the yield strength of actual materials and can accurately predict the yield behavior of thin-film materials. Therefore, its application in nanoindentation testing is significant. The criterion determines whether a material is yielding by calculating a quantity called the Von Mises stress, which is calculated from the principal stress component of the stress tensor, expressed as follows:

(4) $$\sigma_{v}=\sqrt{\frac{1}{2}\left[(\sigma_{1}-\sigma_{2}{)}^{2}+(\sigma_{2}-\sigma_{3}{)}^{2}+(\sigma_{3}-\sigma_{1}{)}^{2}\right]}$$

where $\sigma_{1}$, $\sigma_{2}$, and $\sigma_{3}$ are the principal stress components.

### 3.2. Machine Learning Methods

#### 3.2.1. Database Generation

Machine learning requires a materials database to characterize the behavior of polymers under nanoindentation. This section describes the construction of such a database from relevant material attributes. To ensure a representative and sufficiently covered parameter space, we employ randomized sampling; specifically, Markov Chain Monte Carlo (MCMC) is adopted in this study to explore high-dimensional parameter domains efficiently. By combining Markov chains with Monte Carlo sampling, MCMC generates random samples that asymptotically follow a prescribed target distribution. Owing to its Markov property—the next sample depends only on the current state—sampling can be constrained to the admissible domain, improving coverage where it matters. Consequently, the database enables a more accurate assessment of parameter influences on model performance, while enhancing stability and robustness across varying conditions. The layer-wise parameter ranges to be identified are summarized in Table 4.

**Figure 6:**
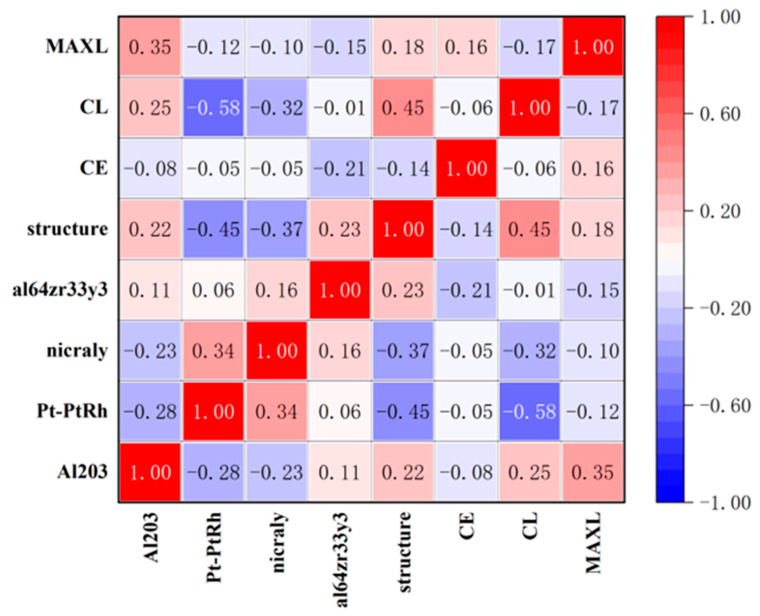
Parameter correlation diagram.

Figure 6 shows 20 sets of parameter combination correlations (σ_v_ (Material name), G_C_ (CE), ∆_C_ (CL), and ∆_Fail_ (MAXL)) generated by the Monte Carlo method. As can be seen from Figure 5, the histogram of the diagonal line reflects the frequency distribution of the values of each variable, which indicates that the distribution characteristics of the samples are reasonable and representative. The thermodynamic diagram of the correlation coefficient in the off-diagonal region shows the linear relationship between variables. It can be observed that the Pt-PtRh plastic parameters of the material show a significant negative correlation with CL (−0.58), and the material Pt-PtRh plastic parameters show a significant negative correlation with CL (−0.58), there was also a negative correlation between the matrix plastic parameters and the Pt-PtRh plastic parameters (−0.4474) and the NICRALY plastic parameters (−0.37), while the MAXL and AL2O3 plastic parameters showed a moderate positive correlation (0.35), it shows that there is a complex interaction between the variables, and there is a complex interaction between the variables.

Then, the material parameter data is input into Marc for calculation, and the calculated indentation curve is extracted as a database. A total of 20 sets of curves were generated as the training database, among which 10% (i.e., 2 sets) were randomly selected and used as the validation dataset.

#### 3.2.2. Machine Learning Algorithm Selection

The model selection and parameters are very important to the accuracy of training in the model training process [45]. The accuracy of the machine learning model of the cross-validation method is carried out by the UserScript algorithm of Odyssee, which can complete the selection of high-precision machine learning algorithms. Following a comparative evaluation of candidate methods, we selected a clustering-based reduced-order modeling approach and Kriging interpolation.

#### 3.2.3. Model Reduction Algorithm

The selected Clustering degree reduction method is effective for the degree reduction of high-dimensional curve data. Its basic idea is to divide a large number of original curves into a small number of clusters according to the similarity of curves, to achieve degree reduction. Specifically, given a dataset of original curves, we first define a similarity measure between any two curves. The commonly used measure is the Euclidean distance between curves:

(5) $$d(X_{i}(t),X_{j}(t))=\sqrt{\int_{t}(X_{i}(t)-X_{j}(t){)}^{2}dt}$$

Based on the above distance measure, the original curve data is divided into *K* clusters by using a hierarchical clustering algorithm, and the center curve of the cluster can represent each cluster:

(6) $$C_{k}(t)=\frac{1}{\left|S_{k}\right|}\sum_{X_{i}(t)\in S_{k}}X_{i}(t),S_{k}=\{X_{i}(t)|X_{i}(t)\in \mathrm{the}\;k-\mathrm{th}\;\mathrm{cluster}\}$$

In this way, the original complex curve data is simplified into several representative center curves, and the effective order reduction of the original curve data space is realized, thus reducing the computational complexity of subsequent data analysis and modeling. At the same time, the data features are well preserved and expressed.

#### 3.2.4. Interpolation Methods

The selected interpolation Kriging method is the optimal linear unbiased estimation interpolation method, which is mathematically proven to minimize the interpolation error under reasonable assumptions. Kriging estimates the value of an unsampled location by weighting and summing the attribute values of the sampling points. The Kriging equations are as follows:

(7) $$\left[\begin{array}{cccc} C(x_{1},x_{1}) & \ldots & C(x_{1},x_{n}) & 1\\ \vdots & \ddots & \vdots & \vdots \\ C(x_{n},x_{1}) & \ldots & C(x_{n},x_{n}) & 1\\ 1 & \ldots & 1 & 0 \end{array}\right]\left[\begin{array}{c} \lambda_{1}\\ \vdots \\ \lambda_{n}\\ -\mu \end{array}\right]=\left[\begin{array}{c} C(x_{0},x_{1})\\ \vdots \\ C(x_{0},x_{n})\\ 1 \end{array}\right]$$

where *x_i_* represents the sampling points, *λ_i_* denotes the weight, *μ* is the Lagrange multiplier, and *C(x,y)* represents the covariance of the observed values. The kriging interpolation method has high interpolation accuracy, can accurately reflect the change in the terrain surface, and can consider the spatial autocorrelation between sample points, so it is suitable for data with strong spatial continuity. At the same time, the smoothness of the interpolation results can be controlled by adjusting the interpolation parameters.

#### 3.2.5. Model Validation

**Figure 7:**
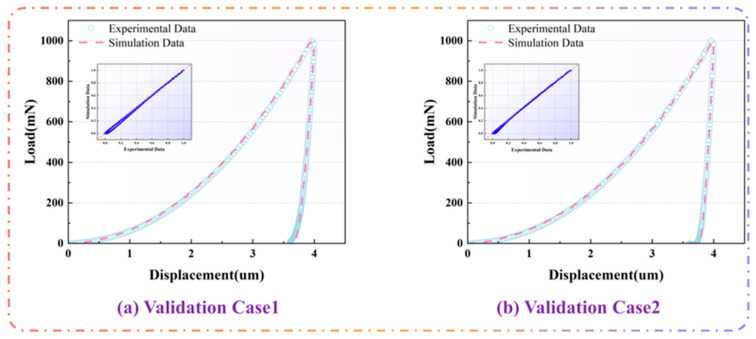
Model validation results.

After the parameters of the model selection results are brought in, the model training results are shown in Figure 6. This time, the data were normalized to a dimensionless range between 0.0 and 1.0 by dividing the value in the Axis by the corresponding maximum value, and the correlation coefficient R^2^ was selected as the Accuracy Indicator. The red dashed line represents the exact agreement between the true and predicted values, i.e., 100% accuracy, where the dashed line is y = x.The model has high prediction accuracy when the data points are mainly concentrated near the Red Line. As shown in Figure 7, the correlation coefficients R^2^ are all at or above 0.99, indicating a high degree of accuracy in the results of the validation curves.

#### 3.2.6. Optimization Model

After the model training was completed, the nanoindentation-fitted experimental load–displacement curve was imported as the target response, and a back-analysis procedure for material parameter optimization was carried out. The optimization algorithm employed was a global, unconstrained, simulation-based optimization method.

In this process, a simulation-driven parameter identification framework was established to calibrate the mechanical parameters of the multilayer thin films. The optimization was performed using the Nelder–Mead simplex method, a classical derivative-free algorithm widely adopted for nonlinear function minimization problems.

The objective function was defined to minimize the deviation between the nanoindentation-fitted experimental curve and the simulated load–displacement response:

(8) $$minF(x)=\sum_{i=1}^{n}{\left[P_{exp}\left(i\right)-P_{sin}(i,x)\right]}^{2}$$

where x represents the material parameters to be identified.

Because the optimization was simulation-driven, each function evaluation required running a finite element analysis (FEA) to compute $P_{\mathrm{sim}}$. The Nelder–Mead method iteratively adjusts a simplex—an n+1-vertex geometric figure in an n-dimensional space—by replacing the worst-performing vertex with an improved one based on reflection, expansion, contraction, and shrink operations. This algorithm does not require gradient information and is therefore well suited for nonlinear, non-smooth, and noisy responses commonly encountered in FEA-based calibration.

No explicit constraints were imposed, as the parameter search domain was physically meaningful and bounded by prior experimental ranges. Convergence was considered achieved when the relative change in the objective function dropped below $10^{-6}$ over 999 consecutive iterations, ensuring numerical stability and robustness.

## 4. Discussion

### 4.1. Effect of Temperature on Material Parameters and Thermal Stability Analysis

Table 5 lists the inversion results of the material parameters of each layer of the multilayer film structure at different temperatures. It can be seen from the analysis that the elastic-plastic mechanical parameters (such as yield strength) and interfacial cohesion model parameters of each layer alloy do not change significantly in the range of 25 °C to 400 °C. This phenomenon is consistent with the actual mechanical behavior of the material at high temperature, indicating that the multilayer film composite structure has excellent thermal stability under high-temperature service conditions. When the temperature rises to 600 °C, most material parameters remain close to room temperature; only the strength of the AL_64_ZR_33_Y_3_ alloy layer and the substrate material decreases slightly, and the cohesive zone model parameters show only a slight fluctuation. In general, the mechanical properties of each layer within 600 °C are not significantly deteriorated, which shows that the structure has good elastic-plastic stability in this temperature range.

**Figure 8:**
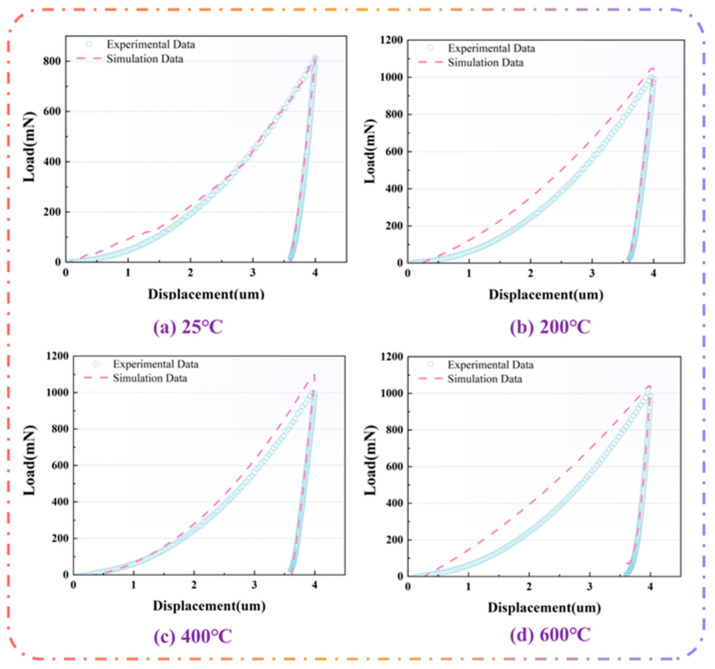
(**a**–**d**) Comparison of experimental and simulated load–displacement curves at different temperatures.

In order to verify the reliability of the parameters, the parameters obtained by the inversion are imported into the Marc finite element platform for numerical simulation, and the calculation results are compared with the experimental results. Figure 8 presents the simulation results. Overall, the simulated and experimental curves agree well: minor deviations appear during the loading stage, whereas the unloading segment matches closely. Using Equations (1) and (2), the hardness and elastic modulus were computed and are summarized in Table 6. As shown, the relative errors with respect to the experimental measurements do not exceed 10% for either quantity, indicating high model accuracy and reliability.

**Figure 9:**
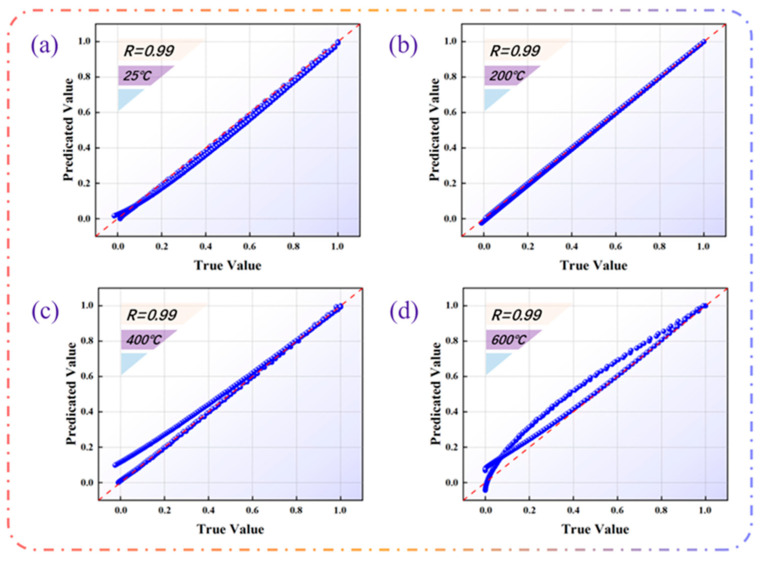
(**a**–**d**) Inverse analysis calculations for different temperature parameters.

As shown in Figure 9, after normalizing the load, the simulated results agree well with the experimental data across all conditions, with coefficients of determination R^2^ reaching or exceeding 0.99. This not only confirms the validity of the constitutive model but also verifies the reliability of the inversely identified parameters.

### 4.2. Efficiency of Back-Analysis

Compared with the traditional nanoindentation inversion methods in the literature, the proposed method has significant advantages in terms of accuracy and efficiency. The calculation time of each model is shown in Table 7. It can be observed from the calculated events that the back analysis of the parameters can be completed in about one hour. The traditional parameter inversion usually relies on the iterative optimization matching process between the finite element simulation and the experimental curve. This process not only needs to consider the relationship between multiple physical quantities at the same time, but also the parametric finite element simulation itself, which is very time-consuming. This means that the reverse analysis method based solely on many finite element simulations is inefficient and has limited applicability to different material systems. In contrast, machine learning methods exhibit unique advantages in dealing with such nonlinear mapping problems: low development cost, short training cycle, and excellent prediction performance on large datasets. Through the machine learning method under the Odyssee platform, this study realizes the efficient mapping of the indentation curve to the material parameters. The indentation response predicted by the model is highly consistent with the experimental curve, and the R^2^ at each temperature point is above 0.99, reaching a nearly perfect fitting degree. This accuracy is better than that reported by most traditional inversion methods. For example, it has been shown that the correlation between the stress field predicted by machine learning and the finite element results can reach R^2^ > 0.99; the nanoindentation inversion model based on the LSTM network can also achieve a prediction accuracy of about 97% [46]. In contrast, the proposed method achieves higher prediction accuracy and stability while maintaining R^2^ ≈ 0.99, highlighting the effectiveness and superiority of the model.

## 5. Conclusions

This study proposes a fast evaluation method for material parameters of multilayer thin-film structures based on machine learning technology, which effectively solves the shortcomings of traditional back analysis methods in terms of time efficiency and result reliability. Detailed mechanical property data were obtained by nanoindentation tests on different layers of thin films, and a two-dimensional elastic-plastic model was constructed by MARC software (version 2024.2) to simulate the nanoindentation process accurately; many indentation test curves were generated. These simulation data are trained on the ODYSSEE platform using machine learning technology, and the rapid reverse analysis of material parameters is realized.

The experimental results show that the proposed method exhibits high accuracy and efficiency in the inversion of interlaminar cohesion parameters, and significantly shortens the time for back analysis of multilayer thin-film structure parameters, the model calculation time is about one h, and the correlation coefficients R all reach or exceed 0.99, which proves the high accuracy of the method in the evaluation of material properties by indentation test parameters. This result not only provides an efficient tool for the performance evaluation of multilayer thin-film materials, but also provides strong support for their optimal design. In addition, this method has broad applicability and great potential in evaluating material parameters of different types of membrane structures.

## Figures and Tables

**Table 1:** Membrane layers of each sample (from bottom to top).

Substrate	Film1 (First Layer)	Film2 (Second Layer)	Film3 (Third Layer)	Film4 (Fourth Layer)
Al_2_O_3_	NiCrAlY	Al_64_Zr_33_Y_3_	PtRh	Al_2_O_3_

**Table 2:** Model dimensions (in mm).

Base	Film1	Film2	Film3	Film4
0.2 × 0.1787	0.2 × 0.016	0.2 × 0.002	0.2 × 0.001	0.2 × 0.0023

**Table 3:** Known Material Parameters.

Material	Density (kg/m^3^)	Elasticity Modulus (Gpa)	Specimen Plate
Al_2_O_3_	3500	344.50	0.29
Pt-PtRh	21,450	200	0.34
Al_64_Zr_33_Y_3_	6000	174	0.37
NiCrAlY	5000	199.90	0.30
Base	8900	219	0.31

**Table 4:** Simulation parameters.

Material	σ*_v_* (Mpa)	$G_{C}$ (mJ/mm^2^)	∆_c_ (mm)	∆^fail^ (mm)
Al_2_O_3_	600–800	800–1000	0.0005–0.001	0.005–0.01
Pt-PtRh	600–800	800–1000	0.0005–0.001	0.005–0.01
Al_64_Zr_33_Y_3_	350–550	800–1000	0.0005–0.001	0.005–0.01
NiCrAlY	500–700	800–1000	0.0005–0.001	0.005–0.01
Base	500–700	800–1000	0.0005–0.001	0.005–0.01

**Table 5:** Parametric inverse analysis results.

T (°C)	NiCrAlY (MPa)	Al_64_Zr_33_Y_3_ (MPa)	Base (MPa)	Al_2_O_3_ (MPa)	Pt-PtRh (MPa)	*G_C_* (mJ/mm^2^)	∆_c_ (mm)	∆^fail^ (mm)
25	613.075	486.158	482.274	770.375	705.088	717.162	0.000798	0.0930
200	613.648	494.365	493.365	779.629	705.184	965.395	0.00858	0.0992
400	613.401	487.925	482.234	781.157	705.895	717.162	0.000863	0.0960
600	613.118	425.897	431.802	769.704	705.255	873.987	0.000695	0.0539

**Table 6:** Nanoindentation Curve Calculation Results.

T (°C)	Experiment		Simulation	
	H (GPa)	E (GPa)	H (GPa)	E (GPa)
25	2.86	125.65	2.83	113.72
200	2.93	135.59	3.02	135.22
400	3.15	138.00	2.96	146.11
600	2.35	189.46	2.29	207.95

**Table 7:** Back analysis calculation time of nanoindentation curves at various temperatures.

T (°C)	25	200	400	600
Time (min)	61	72	52	64

## Data Availability

The original contributions presented in this study are included in the article. Further inquiries can be directed to the corresponding author.
